# A prospective multicenter study of direct comparison of feasibility and safety of pulmonary vein isolation using the minimally interrupted apixaban between second‐generation cryoballoon and radiofrequency ablation of paroxysmal atrial fibrillation: J‐HIT apixaban

**DOI:** 10.1002/joa3.12392

**Published:** 2020-06-27

**Authors:** Atsuhiko Yagishita, Masahiko Goya, Yoshito Iesaka, Junichi Nitta, Atsushi Takahashi, Yasutoshi Nagata, Hitoshi Hachiya, Osamu Inaba, Yukihiro Inamura, Yasuaki Tanaka, Keita Watanabe, Susumu Tao, Yasuhiro Shirai, Tasuku Yamamoto, Shinya Shiohira, Kikou Akiyoshi, Masahiro Sekigawa, Shingo Maeda, Takeshi Sasaki, Yoshihide Takahashi, Mihoko Kawabata, Kenzo Hirao, for the J‐HIT investigators

**Affiliations:** ^1^ Department of Cardiovascular Medicine/Heart Rhythm Center Tokyo Medical and Dental University Tokyo Japan; ^2^ Cardiovascular Center Tsuchiura Kyodo Hospital Tsuchiura Japan; ^3^ Department of Cardiology Saitama Red Cross Hospital Saitama Japan; ^4^ Cardiovascular Center Yokosuka Kyosai Hospital Yokosuka Japan; ^5^ Division of Cardiology Musashino Red Cross Hospital Tokyo Japan; ^6^ Department of Advanced Arrhythmia Research Tokyo Medical and Dental University Tokyo Japan

**Keywords:** apixaban, atrial fibrillation, catheter ablation

## Abstract

**Background:**

The feasibility and safety of pulmonary vein isolation (PVI) using cryoballoon (CB) for paroxysmal atrial fibrillation (PAF) with minimally interrupted apixaban has not fully explored.

**Methods:**

In this multicenter, randomized prospective study, we enrolled patients with PAF undergoing CB or radiofrequency (RF) ablation with interrupted (holding 1 dose) apixaban. The primary composite end point consisted of bleeding events, including pericardial effusion and major bleeding requiring blood transfusion, or thromboembolic events at 4 weeks after ablation; secondary end points included early recurrence of AF and procedural duration.

**Results:**

A total of 250 patients underwent PVI (125 assigned to the RF ablation and 125 assigned to the CB ablation). The primary end point occurred in 1 patient in the CB ablation group (0.8%; 90% confidence interval [CI], 0.04 to 3.70) and 3 patients in the RF group (2.4%, *P* = .622; risk ratio, 0333; 90% CI, 0.05 to 2.20). All events were pericardial effusion, all of whom recovered after pericardiocentesis. Early recurrence of AF occurred in 4 patients (3.2%) in the RF group and in 6 patients (4.8%) in the CB group (*P* = .749). The procedural duration was shorter in the CB group than that in the RF group (136.5 ± 39.9 vs 179.5 ± 44.8 min, *P* < .001).

**Conclusion:**

CB ablation with minimally interrupted apixaban was feasible and safe in patients with PAF undergoing PVI, which was equivalent to RF ablation.

## Graphical abstract

Comparison of Safety and Feasibility of Apixaban between Cryoballoon and Radiofrequency pulmonary vein isolation for paroxysmal atrial fibrillation

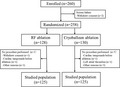

**Abbreviations**
CB: cryoballoon
PAF: paroxysmal atrial fibrillation
RF: radiofrequency

## 1 INTRODUCTION

Catheter ablation is a well‐established therapy in patients with symptomatic drug‐refractory paroxysmal atrial fibrillation (PAF), but it is one of the most complex interventional electrophysiological procedures and associated with a significant risk of complications, such as thromboembolic or bleeding events.^1^ Uninterrupted warfarin during the periprocedural period has been used as a standard protocol to minimize the risk of thromboembolic events.^2^, ^3^ Direct oral anticoagulants (DOACs) are increasingly used in patients undergoing catheter ablation. Evidence for the safety of uninterrupted DOACs has increased with the recent publication of Venture AF, RE‐CIRCUIT, and AXAFA compared with uninterrupted warfarin.^4^, ^5^, ^6^ Recently, Reynolds et al have demonstrated the low rate of thromboembolic and bleeding events with the use of minimally interrupted apixaban compared with uninterrupted apixaban and warfarin.^7^

Pulmonary vein isolation (PVI) is a cornerstone of catheter ablation, particularly for PAF. Cryoballoon (CB) ablation has emerged as an alternative technique to radiofrequency (RF) ablation for the treatment of PAF. The recent largest randomized trial comparing both technologies in patients with PAF demonstrated the noninferiority of CB ablation to RF ablation in terms of efficacy and safety end points.^8^ To date, there has been no prospective study to determine the feasibility and safety of CB ablation using DOACs in comparison with those of RF ablation. We hypothesized that the safety and efficacy of minimally interrupted apixaban in patients undergoing CB ablation is noninferior to those receiving RF ablation. In this multicenter, prospective study (Japan Heat and Ice Trial [J‐HIT]), we sought to determine the feasibility and safety of CB ablation with minimally interrupted apixaban in patients with PAF.

## 2 MATERIALS AND METHODS

### 2.1 Study Design

The J‐HIT was a multicenter, pilot randomized, controlled trial (RCT) to assess the feasibility and safety of CB ablation in comparison with RF ablation. The aims of the trial were 2‐folds. First, we aimed to show that CB ablation with minimally interrupted apixaban was feasible and safe by comparing to the predefined threshold (noninferiority of CB ablation). Second, we aimed to compare the feasibility and safety of CB and RF ablation prospectively (exploratory comparison of CB and RF ablation). The trial was investigator initiated; the steering committee was responsible for the design, execution, and conduct of the study. The institutional review board approved the study. This study has been registered at the UMIN Clinical Trial Registry (number UMIN000018901). A data‐ and safety‐monitoring board reviewed interim results and monitored the safety of the patients. The members of an end point review committee, who were unaware of the treatment‐group assignments, adjudicated primary safety events. All members of the steering committee approved the statistical analysis and interpretation of the data. The trial was funded by Bristol‐Myers Squibb. A contract research organization (Clinical Research Support Center, The University of Tokyo Hospital, Tokyo, Japan) collected, monitored, maintained, and analyzed the data.

### 2.2 Study participants

Five Japanese centers participated in the trial, and patients aged ≥18 and ≤85 years who had symptomatic PAF refractory to class I or class III antiarrhythmic drugs or *β*‐blockers were enrolled. Patients were mentally and linguistically able to understand the aim of the trial and to show sufficient compliance in the trial protocol and agree to these risks and benefits as stated in the patient informed consent document. All patients have signed the informed consent form for the trial and were randomly assigned, in a 1:1 ratio, to the CB ablation (CB group) or RF ablation (RF group). Patients with mechanical heart valves, advanced hepatic or renal (creatinine clearance <15 mL/min or on dialysis) dysfunction, any condition contraindicating chronic anticoagulation including hypersensitivity to apixaban or bleeding disorders, active systemic infection, pregnant or breastfeeding women, or women of childbearing potential not on adequate birth control were excluded.

### 2.3 Catheter ablation procedure and anticoagulation protocol

Patients were off antiarrhythmic drugs for at least 5 times the drugs’ half‐lives before the ablation. Transesophageal echocardiography was performed in all patients within 24 hours before the procedure. All patients were treated with apixaban for at least 4 weeks before the catheter ablation. Apixaban was administered at a dose of 5 or 2.5 mg 2 times daily, and the latter dose was provided to the patients who met 2 or more of the following criteria: age ≥ 80 years, body weight ≤ 60 kg, or serum creatinine level of ≥1.5 mg/dL. Apixaban was held at 1 dose before the procedure, and administration resumed after the procedure. All patients had a groin‐entry venous‐route catheter introduction with entry into the left atrium via transseptal puncture. The activated clotting time was maintained at 300‐350 seconds with heparinized‐saline infusion. The CB catheter was introduced into and maneuvered within the left atrium through a 12Fr steerable sheath (FlexCath^®^; Medtronic, Inc) using an integrated circular mapping catheter (Achieve^™^; Medtronic, Inc). Operators attempted PVI by placing the device under fluoroscopic guidance at each pulmonary vein antrum, advancing it toward the PV to achieve occlusion, and then cooling the tissue by filling the balloon with a liquid refrigerant. Ablation lesions with the CB system were created using intracatheter temperatures of about –50°C delivered to each PV. Second‐generation CB was used in this study (Arctic Front Advance^®^; Medtronic, Inc). In general, each CB ablation was performed with a target ablation time of 180 seconds. During right‐sided ablations, phrenic nerve function was manually monitored by palpitation with the aid of diaphragmatic pacing from the superior vena cava. Freezing was immediately truncated if diaphragmatic weakness or palsy occurred, and no further CB ablation was performed at the respective PV. RF ablation was performed using a 3.5‐mm irrigated open‐tip contact force‐sensing catheter or 8‐mm nonirrigated catheter. A point‐by‐point series was used to encircle the left‐ and right‐sided PVs. In general, power settings included that power should not exceed 40 and 30 W at the anterior and posterior aspect, respectively, with an RF duration of 30 seconds. The amount of contact force applied was left to the discretion of the operators. The acute procedural end point was defined as the absence of all PV potentials, as confirmed by bidirectional block using a circular mapping catheter. Use of a 3‐dimensional electroanatomical mapping system, including CARTO system (Biosense Webster, Inc) or Ensite Velocity system (Abbott), was at the discretion of operators. In all patients, the aim was to completely isolate the PVs. Therefore, touch‐up ablation for complete isolation was performed if deemed necessary. No additional left atrial ablation was allowed. Patients resumed apixaban at their usual dose in the evening after the procedure and continued for 4 weeks postprocedure.

### 2.4 Patient follow‐up and study end points

After the procedure, a follow‐up visit was scheduled for 4 weeks. At the visit, a medical history was obtained, a physical examination was performed, and a 12‐lead electrocardiogram was completed. The primary end point was a composite of thromboembolic or bleeding events at 4 weeks postprocedure: cerebral stroke, transient ischemic attack, pericardial effusion requiring pericardiocentesis, hematoma or bleeding events requiring blood transfusion, and intracranial hemorrhage. The prespecified secondary end points included early recurrence of AF, minor bleeding recovered without blood transfusion, pericardial effusion not requiring pericardiocentesis, and total duration of the procedure. Early recurrence of AF was defined as the documented recurrence of AF lasting more than 30 seconds.

### 2.5 Statistical analysis

In this pilot RCT, we first aimed to show single‐arm, noninferiority of the CB ablation with apixaban. The reported incidence of thromboembolic or major bleeding in patients undergoing RF ablation of AF with apixaban was 2%.^9^ According to the results from German ablation registry, there was no difference in the complication rate between CB and RF ablation with phenprocoumon, a derivative of coumarin.^10^ In the ARISTOTLE study, the rate of stroke or systemic embolism of apixaban was not inferior to that of warfarin of 1.60% per year. In this study, we defined the noninferiority threshold of a complication rate of 5%. When the incidence and upper limit of 90% confidence interval (CI) of complications are 1.60% and 5%, respectively, we calculated that 125 patients or more would have to be enrolled. Since our second aim was to compare RF and CB ablation prospectively, we chose to randomize patients as 1:1 ratio. Therefore, the target sample size was 260 patients in total consisting of 130 patients each for RF ablation and CB ablation in this study Continuous variables and percentages for categorical variables are expressed as mean ± standard deviation. A comparison between the groups was performed with the Student *t* test or the Wilcoxon rank‐sum test, as appropriate. For categorical data, Fisher's exact test was applied. A 2‐tailed *P* value of <.05 was considered to indicate statistical significance. Analyses were conducted with SAS software, version 9.4 (SAS Institute).

## 3 RESULTS

### 3.1 Patient enrollment and characteristics

**FIGURE 1:**
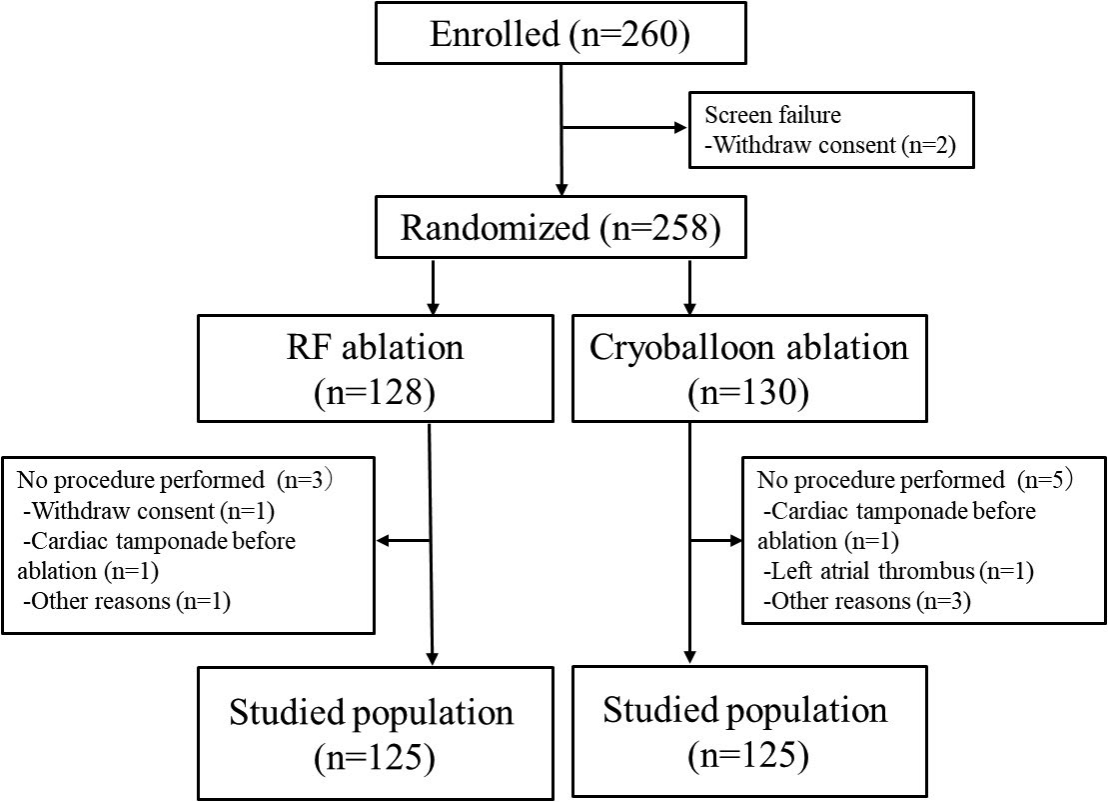
Disposition of patients in the prospective cohort. Disposition of the 260 enrolled patients, 250 of whom were included in the evaluable population, is shown

A total of 260 patients were enrolled (Figure 1). After enrollment, 2 patients withdrew consent, and the remaining 258 patients were assigned to a treatment group (130 in the CB group and 128 in the RF group). In the CB group, 5 patients withdrew from the trial because of a left atrial thrombus on preprocedural transesophageal echocardiography, cardiac tamponade before left atrial ablation, use of another anticoagulant, and procedures before consent in 2 patients. In the RF group, 3 patients withdrew from the trial because of withdrawal of consent, cardiac tamponade before left atrial ablation, and a procedure before consent. In the remaining 250 patients (125 in the CB group and 125 in the RF group), there was no difference in baseline patient characteristics between the 2 groups (Table 1). One‐hundred and ninety‐five of the 250 patients (78%) underwent computed tomography before the ablation procedures. Anatomical variants were found in 40 of the 195 patients (21%), including the left common PVs in 15 patients (CB 11, RF 4), the right middle PVs in 11 patients (CB 6, RF 5), both the left common and the right middle PVs in 4 patients (CB 2, RF2), the LA roof veins in 4 patients (CB1, RF 3), the anterior LA diverticulums in 5 patients (CB 4, RF1), and the inferior common PV in 1 patient (RF). In the 125 patients undergoing the RF ablation, CARTO system was used in 81 patients (65%), whereas Ensite Velocity system was used in the remaining 44 patients. Complete isolation was achieved in all patients in both groups. Of the 125 patients undergoing the CB ablation, touch‐up ablation performed in 25 patients (20%), including 4 LSPVs, 5 LIPVs, 2 left common PVs, 4 RSPVs, 16 RIPVs, and 1 right middle PV.

**TABLE 1:** Baseline demographic and clinical characteristics

	Total (N = 250)	Radiofrequency ablation (n = 125)	Cryoballoon ablation (n = 125)	*P* value
Age	63.1 ± 10.5	64.2 ± 9.8	62.0 ± 11.1	.075
Male (%)	182 (72.8)	94 (75.2)	88 (70.4)	.478
BMI (kg/m^2^)	24.01 ± 3.38	24.04 ± 3.36	23.97 ± 3.41	.892
History of paroxysmal AF (months)	46.2 ± 60.6	54.0 ± 68.2	38.4 ± 51.0	.214
Heart failure (%)	2 (0.8)	2 (1.6)	0	.498
Hypertension (%)	126 (50.4)	63 (50.4)	63 (50.4)	.000
Diabetes mellitus (%)	24 (9.6)	9 (7.4)	15 (12.0)	.283
Prior stroke (%)	6 (2.4)	4 (3.2)	2 (1.6)	.684
Ischemic heart disease (%)	12 (4.8)	6 (4.8)	6 (4.8)	.000
CHADS_2_ score	0.8 ± 0.8	0.8 ± 0.8	0.8 ± 0.8	.907
CHA_2_DS_2_‐VASc score	1.6 ± 1.3	1.6 ± 1.3	1.5 ± 1.3	.372
LVDd (mm)	46.2 ± 5.0	46.3 ± 5.2	46.1 ± 4.8	.843
LVEF (%)	66.2 ± 8.2	65.9 ± 9.5	66.5 ± 6.6	.466
LAD (mm)	36.7 ± 6.2	37.1 ± 6.3	36.3 ± 6.2	.348
BNP (pg/mL)	53.12 ± 66.45	55.27 ± 62.50	50.94 ± 70.42	.549
PT (s)	12.61 ± 1.77	12.65 ± 1.76	12.57 ± 1.79	.782
APTT (s)	33.56 ± 4.30	33.90 ± 4.67	33.23 ± 3.90	.241
D‐dimer (μg/mL)	0.28 ± 0.87	0.22 ± 0.26	0.33 ± 1.21	.330
Left appendage flow (m/s)	0.68 ± 0.24	0.656 ± 0.245	0.708 ± 0.239	.217

Abbreviations: AF, atrial fibrillation; APTT, activated partial thromboplastin time; BMI, body mass index; BNP, brain natriuretic peptide; LAD, left atrial diameter; LVDd, left ventricular end‐diastolic dimension; LVEF, left ventricular ejection fraction; PT, prothrombin time.

### 3.2 Efficacy and safety end points

The primary composite end point occurred in 1 patient in the CB group (0.8%; 90% CI, 0.04 to 3.70) and 3 patients in the RF group (2.4%), respectively (risk ratio, 0.333; 90% CI, 0.05 to 2.20; *P* = .622; Table 2). All 4 primary events were pericardial effusion requiring pericardiocentesis (1.6% of 250 patients). There were no cases of cerebral stroke, transient ischemic attack, hematoma or bleeding events requiring blood transfusion, or intracranial hemorrhages.

**TABLE 2:** Efficacy and safety end points

	Radiofrequency ablation（n = 125）	Cryoballoon ablation（n = 125）	*P* value	Risk ratio [90% CI]
Primary end point				
Composite end point	3 (2.4) [0.70 to 6.10]	1 (0.8) [0.04 to 3.70]	0.622	0.333 [0.05 to 2.20]
Pericardial effusion	3 (2.4) [0.70 to 6.10]	1 (0.8) [0.04 to 3.70]	0.622	0.333 [0.05 to 2.20]
Major bleeding	0	0		
Thromboembolic events	0	0		
Secondary end point				
AF recurrence	4 (3.2) [1.1 to 7.2]	6 (4.8) [2.1 to 9.3]	0.749	1.500 [0.53 to 4.25]
Minor bleeding	4 (3.2) [1.1 to 7.2]	4 (3.2) [1.1 to 7.2]	1.000	1.000 [0.32 to 3.14]

Note

Values are n (%) [90% confidence interval].

Abbreviation: AF, atrial fibrillation; CI, confidence interval.

**FIGURE 2:**
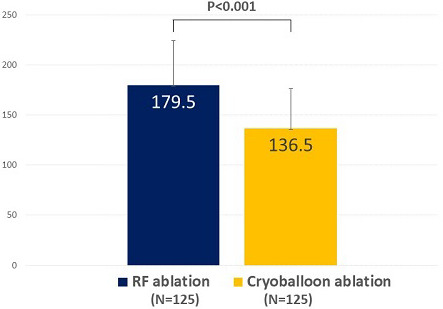
Procedural duration. Comparison of the procedural duration between radiofrequency and cryoballoon ablation. Note the shorter procedural duration in patients undergoing cryoballoon ablation

The secondary efficacy end points are also shown in Table 2. Early recurrence of AF occurred in 4 patients (3.2%) in the RF group and 6 patients (4.8%) in the CB group (*P* = .749). Minor bleeding occurred in 4 patients (3.2%) in the RF group and 4 patients (3.2%) in the CB group (*P* = 1.000). All events were groin hematomas, which recovered without surgeries. The mean total procedure time was shorter in the CB group than that in the RF group (136.5 ± 39.9 vs 179.5 ± 44.8 minutes, *P* < .001, Figure 2).

## 4 DISCUSSION

This multicenter prospective pilot RCT examined the feasibility and safety of minimally interrupted apixaban during the CB and RF ablation of PAF. Our major findings include the following: (a) CB ablation was feasible and safe in comparison with the predefined threshold; (b) there was no difference in terms of the primary composite end point between the 2 groups, and all events of the primary end points were pericardial effusion requiring pericardiocentesis in both groups; (c) the incidences of early recurrence of AF and minor bleeding were similar between the 2 groups; and (d) the CB ablation procedure time was shorter than that of RF ablation.

### 4.1 CB vs RF ablation

In this study, there were no thromboembolic events at 4 weeks postprocedure, suggesting the efficacy of minimally interrupted apixaban as an optimal anticoagulation method in both the RF and CB ablation groups. Pericardial effusion was the only bleeding event, and the incidence was 2.4% in the RF group and 0.8% in the CB group. The reported incidence of pericardial effusion in patients undergoing PVI using a contact force‐sensing catheter was 2.5%,^11^ whereas it was 1.5% in patients undergoing CB ablation.^12^ In the FIRE AND ICE trial, a prospective randomized comparison of the efficacy between CB and RF ablation, the incidence of pericardial effusion was 1.3% in the RF ablation group and 0.3% in the CB ablation group.^8^ A recent registry showed that the incidence of pericardial effusion was similar between CB and RF ablation groups, but it was slightly higher in patients undergoing RF ablation (1.3% vs 0.8%, *P* = .261).^13^ In a meta‐analysis comparing the efficacy and safety of CB vs RF ablations, the incidence of cardiac tamponade was significantly higher in the RF ablation group.^14^ The relatively high incidence of cardiac tamponade in the RF group in comparison with the CB group in our study was consistent with these results. Although the results of our study were similar to those of these previous studies, the lack of previous randomized comparisons between the 2 technologies under the same anticoagulation methods using DOACs highlights the novelty of J‐HIT apixaban.

The incidence of minor bleeding (3.2%) in our study was similar to that in the FIRE AND ICE trial (4.3%).^8^ In our study, there was no difference in the early recurrence of AF across the 2 technologies (3.2% in the RF group and 4.8% in the CB group), which was also consistent with the results in the FIRE AND ICE trial (2.7% in the RF ablation group vs 0.8% in the cryoablation group, *P* = .09). Phrenic nerve injury has been recognized as a common complication, particularly with CB ablation.^1^, ^14^ There were no phrenic nerve injuries in this study, possibly because of the operator's experience associated with the adequate hospital procedure volume among the centers. The procedure duration was shorter in the CB group, which was consistent with the results of previous reports,^8^, ^13^, ^15^, ^16^, ^17^, ^18^, ^19^, ^20^ likely because of the shorter duration required for isolating each PV by a single‐step circumferential lesion with CB ablation than that by a point‐by‐point circumferential lesion with RF ablation.

### 4.2 Minimally interrupted vs uninterrupted apixaban

Uninterrupted warfarin has been a standard anticoagulation therapy during the periprocedural period of catheter ablation. Recent studies have demonstrated that the use of uninterrupted or minimally interrupted DOACs during catheter ablation of AF was noninferior to the use of uninterrupted warfarin in terms of the thromboembolic and bleeding events.^1^ Literature specific to apixaban also described favorable results with other DOACs.^4^, ^9^, ^21^, ^22^, ^23^, ^24^, ^25^ One prospective study showed that uninterrupted apixaban was safe and effective compared with uninterrupted warfarin.^4^ The recent AEIOU trial investigated the utility of minimally interrupted apixaban in comparison with uninterrupted apixaban.^7^ This prospective, multicenter trial randomized 300 patients to uninterrupted vs minimally interrupted periprocedural apixaban, and the results showed no stroke or thromboembolic events at 4 weeks after ablation and no difference in bleeding events between the 2 anticoagulation strategies. In the AEIOU study, 30% of the patients underwent CB ablation, but efficacy and safety were not compared between the different ablation technologies. In the J‐HIT study, which collected data from 5 Japanese institutes, the addition of apixaban added unique data to the randomized comparison between CB and RF ablation, in terms of the outcomes of minimally interrupted apixaban.

### 4.3 Limitations

First, this is a pilot RCT and sample size may be small to demonstrate the noninferiority in head‐to‐head comparison of these methods. Second, we did not routinely perform brain magnetic resonance imaging to search for new brain lesions after the ablation, so asymptomatic thromboembolic events may have occurred. Third, neither left atrium dwell time nor fluoroscopic time during the procedure were assessed in this study. In addition, PV occlusion scores, time to PVI, duration of the freezing procedure, and number of freezes were not recorded in the CB ablation group. Fourth, neither the number of RF applications nor the contact force measurements were recorded in the RF ablation group.

## 5 CONCLUSIONS

In this pilot RCT, CB ablation with minimally interrupted apixaban was feasible and safe in patients with PAF undergoing PVI by comparing to the predefined threshold, and CB and RF ablation was equally feasible and safe.

## DISCLOSURE

The local institutional review board of Tokyo Medical and Dental University approved the trial on June 23, 2015 (number R2015‐012), and the trial was registered at the UMIN Clinical Trial Registry on September 3, 2015 (number UMIN000018901). Drs. Takahashi and Maeda received an endowment from Medtronic, Boston Scientific, Japan Lifeline, and WIN International. All the other authors report that they have no relationships to disclose that are relevant to the contents of this paper.

## Supporting information

Supplementary Material
