# Supplementary material for: A prospective multicenter study of direct comparison of feasibility and safety of pulmonary vein isolation using the minimally interrupted apixaban between second‐generation cryoballoon and radiofrequency ablation of paroxysmal atrial fibrillation: J‐HIT apixaban
Source: J Arrhythm. 2020 Jun 27;36(4):617–23. doi: 10.1002/joa3.12392 (PMC7411193; doi:10.1002/joa3.12392)
Supplement: Supplementary file 1 — Supplementary Material [file JOA3-36-617-s001.docx]

**Appendix 1. Committees and Investigators**

**Steering Committee**

Kenzo Hirao; Masahiko Goya; Yoshito Iesaka; Junichi Nitta; Atsushi Takahashi; Yasutoshi Nagata.

**Investigators**

**Department of Cardiovascular Medicine / Heart Rhythm Center, Tokyo Medical and Dental University, Tokyo, Japan** Tasuku Yamamoto; Shinya Shiohira; Kikou Akiyoshi; Masahiro Sekigawa; Yasuhiro Shirai; Atsuhiko Yagishita; Susumu Tao; Takeshi Sasaki; Mihoko Kawabata.

**Department of Advanced Arrhythmia Research, Tokyo Medical and Dental University, Tokyo, Japan** Shingo Maeda; Yoshihide Takahashi.

**Cardiovascular Center, Tsuchiura Kyodo Hospital, Tsuchiura, Japan** Satoshi Hara; Yoshikazu Sato; Kazuya Yamao; Shigeki Kusa; Hitoshi Hachiya.

**Department of Cardiology, Saitama Red Cross Hospital, Saitama, Japan** Giichi Nitta; Toshikazu Kono; Takashi Ikenouchi; Kazuya Murata; Tatsuhiko Hirao; Yukihiro Inamura; Tomomasa Takamiya; Osamu Inaba.

**Cardiovascular Center, Yokosuka Kyosai Hospital, Yokosuka, Japan** Yosuke Hayashi; Takamitsu Takagi Jun Nakajima Yasuaki Tanaka.

**Division of Cardiology Musashino Red Cross Hospital, Tokyo, Japan** Junji Yamaguchi; Keita Watanabe; Yuichiro Sagawa; Masakazu Kaneko; Naoyuki Miwa.
